# Moxibustion treatment for diarrhea-predominant irritable bowel syndrome: study protocol for a randomized controlled trial

**DOI:** 10.1186/s12906-016-1386-4

**Published:** 2016-10-24

**Authors:** Chunhui Bao, Jingzhi Zhang, Jinmei Liu, Huirong Liu, Luyi Wu, Yin Shi, Jing Li, Zhihai Hu, Yongzheng Dong, Siyao Wang, Xiaoqing Zeng, Huangan Wu

**Affiliations:** 1 Key Laboratory of Acupuncture and Immunological Effects, Shanghai University of Traditional Chinese Medicine, 650 South Wanping Road, Shanghai, 200030 China; 2 Outpatient Department, Shanghai Research Institute of Acupuncture and Meridian, Shanghai University of Traditional Chinese Medicine, Shanghai, 200030 China; 3 Department of Acupuncture-Moxibustion, Yueyang Integrated Chinese and Western Medicine Hospital, Shanghai University of Traditional Chinese Medicine, Shanghai, 200437 China; 4 Department of Acupuncture-Moxibustion, Shanghai Traditional Chinese Medicine-Integrated Hospital, Shanghai University of Traditional Chinese Medicine, Shanghai, 200082 China; 5 Department of Gastroenterology, Zhongshan Hospital, Fudan University, Shanghai, 200032 China

**Keywords:** Diarrhea, Irritable bowel syndrome, Moxibustion, Randomized controlled trial

## Abstract

**Background:**

Irritable bowel syndrome (IBS) is a worldwide disease with high morbidity. The effect of current treatment with Western medicine is not satisfactory. Although moxibustion treatment is widely used for gastrointestinal diseases, randomized controlled trials on the use of this treatment for IBS are limited. This study aims to evaluate the clinical efficacy and safety of moxibustion treatment in patients with diarrhea-predominant irritable bowel syndrome (IBS-D).

**Methods/design:**

A multi-center, randomized, single-blind and placebo-controlled trial is employed. 104 cases will be divided into two groups: (1) a mild-warm moxibustion group in which moxa stick is 3–5 cm away from acupuncture points and the skin temperature is maintained at 43 ± 1 °C; and (2) a placebo moxibustion group in which moxa stick is 8–10 cm away from acupuncture points and the skin temperature is maintained at 37 ± 1 °C. Moxibustion is performed on bilateral ST25 and ST36 in the two groups for 30 min each time, three times a week for 6 weeks. The patients are followed up at the 12th and 18th weeks. Adequate relief is used as a primary outcome measure; IBS symptom severity score, Bristol stool form scale, IBS quality-of-life questionnaire, and hospital anxiety and depression scale are used as secondary outcome measures.

**Discussion:**

This study aims to demonstrate the safety and efficacy of moxibustion treatment for IBS-D, which may validate moxibustion as an effective therapy for treating IBS-D.

**Trial registration:**

ClinicalTrials.gov identifier NCT02421627 (8 April 2015).

## Background

Irritable bowel syndrome (IBS) is a chronic functional gastrointestinal disorder characterized by recurrent abdominal pain or discomfort in combination with disturbed bowel habits in the absence of an identifiable cause [1]. Currently, the morbidity of IBS is 7–21 % worldwide, it can occur at any age, mainly between 30 and 50 years [2, 3]. Of all types, diarrhea-predominant irritable bowel syndrome (IBS-D) is the most common subtype [4]. IBS has gained attention because it significantly reduces people’s quality of life (QOL) and influences medical resources and social economy [5, 6].

The pathogenesis of IBS is unclear. It is probably related to visceral hypersensitivity, brain–gut regulation dysfunction, intestinal infection and inflammation, and psychological factors [2, 7]. The current treatment for IBS is to remove the inducing factors and relieve the symptoms. However, the poor efficacy and the side effects from long-term drug use have forced patients to seek alternative interventions [8, 9]. In recent years, physicians as well as patients are more interested in complementary and alternative medicine including acupuncture and moxibustion [10, 11]. While much less popular than acupuncture, moxibustion exerts its effects through warm stimulation caused by burning moxa on the acupuncture points. Mild-warm moxibustion is a therapy in which a burning moxa stick is pointed to a moxibustion site (usually an acupuncture point) at a distance to make the patient have a warm sensation but without any burning pain. Because of its low cost and easy application, moxibustion is often used to treat gastrointestinal diseases. However, its efficacy on IBS has not been confirmed. Previous trials on the treatment of IBS with moxibustion were often lack of strict control of randomization and related risk of bias, which makes the conclusions less convincing [12]. A recent randomized controlled trial on herb-partitioned moxibustion for IBS also failed to fully confirm its effectiveness, and the results still need to be verified [13].

Therefore, a multi-center, parallel-group, 1:1 allocation, randomized, single-blind and placebo-controlled trial has been designed in this study. The aim is to evaluate the clinical efficacy and safety of moxibustion in patients with IBS-D. The results may provide evidence for the clinical use of moxibustion in patients with IBS-D.

## Methods/design

### Study design

**Fig. 1:**
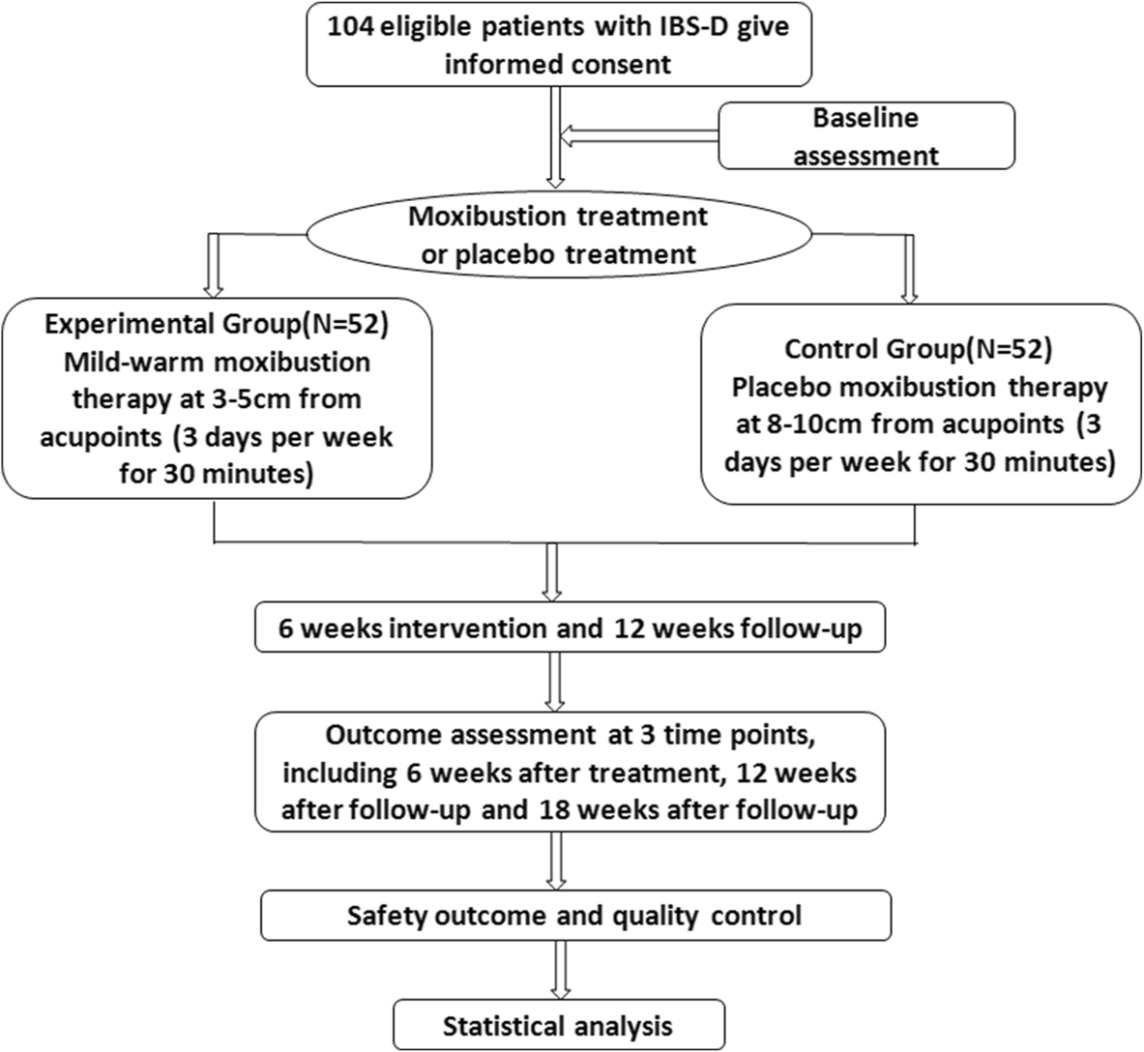
Flow diagram of study design

This study is a multi-center, randomized, single-blind and placebo-controlled clinical trial. All the subjects will be enrolled according to the ROME III IBS-D diagnostic criteria [14]. The flowchart of the trial is shown in Fig. 1.

### Patients

All the 104 cases will be recruited from the following three research centers: the Bowel Disease Clinic of the Shanghai Research Institute of Acupuncture and Meridian; the Department of Acupuncture-Moxibustion of the Yueyang Integrated Chinese and Western Medicine Hospital at the Shanghai University of Traditional Chinese Medicine (TCM); and the Department of Acupuncture-Moxibustion of the Shanghai TCM-Integrated Hospital, Shanghai University of TCM. Recruitment notices were released on TV, newspapers, and magazines. Also, recruiting posters were posted in the community and public places nearby to ensure enough subjects.

### Sample size calculation

Previous studies showed that the effective rates of the mild-warm moxibustion group and the placebo group were 88.7 % [15] and 62 % [16], respectively. Based on these values, the sample size in this study is estimated as follows:

$$ n = {\left(Ua+Ub\right)}^2\left(1 + 1/k\right)P\left(1-P\right)/{\left(Pe-Pc\right)}^2 $$

The significant level is 0.05; the power of test is 1 − *β* = 0.9, *k* = 1; and the sample loss rate is 15 %. The required sample size is 52 by calculation, and no less than 104 cases should be included in the two groups.

### Randomization

In this study, random numbers are generated by SPSS and kept by a clinical research coordinator who has no contact with the patients. The allocation will be concealed in sequentially numbered, opaque, sealed envelopes containing the randomization assignments. An eligible subject who meets the inclusion criteria will sign the informed consent. Researchers in a research center will use a random number and grouping information sent by telephone, text message, or e-mail from the clinical research coordinator, and assign the subject into moxibustion or placebo moxibustion groups.

### Blinding

The practitioners could not be blinded due to the unique nature of moxibustion treatments. The patients are blinded to the treatment. They are kept in individual isolated rooms, avoiding communication among them. Furthermore, Researchers responsible for data collection, analysis, and statistics have no information about grouping and treatment.

### Eligibility

Inclusion criteria:

- Patients who are enrolled according to the ROME III IBS-D diagnostic criteria
- Patients aged between 18 and 65 years
- Patients who have signed informed consent

Exclusion criteria:

- Patients who present with intestinal organic disorders
- Patients who have been administered drugs for treating IBS-D or other drugs that may influence the trial
- Patients who have a severe disease of the heart, brain, lung, liver, kidney, or hematopoietic system
- Patients who are diagnosed with a psychiatric disorder
- Women in pregnancy or nursing
- Patients who have a history of abdominal surgery
- Patients who have been treated by acupuncture and moxibustion

### Interventions

#### Treatment group

For mild-warm moxibustion treatment, bilateral ST25 and ST36 points are used (reference for PRC National Standard GB/T 12346–2006) [17]. A moxa stick (diameter 3 cm) is ignited and positioned with a supporting device so that the tip of the moxa stick is 3–5 cm above the acupuncture point. The temperature of the acupuncture point is monitored with an infrared thermometer (Fluke 62, USA) and maintained at 43 ± 1 °C to make the subject feel warm without any burning pain. The treatment lasts for 30 min each time, three times a week for 6 weeks (18 times). After the treatment, follow-ups are made at weeks 12 and 18.

#### Control group

For the placebo moxibustion group, the same acupuncture points as the treatment group are used. The same type of moxa stick is ignited but kept 8–10 cm above the acupuncture point. The temperature of the acupuncture point is similarly monitored but maintained at 37 ± 1 °C so that the subjects do not to feel warm at the points. The treatment cycle and follow-up time are the same as the treatment group.

### Outcome measurements

#### Primary outcome

##### Adequate relief

Adequate relief (AR) is used to evaluate the degree of alleviation of IBS symptoms, with relief of abdominal pain or abdominal discomfort as the criterion. During the treatment and follow-up, the patients evaluate themselves once every week. The primary endpoint is set at the end of the treatment (week 6), and the statistics are also performed during follow-ups (weeks 12 and 18) for efficacy duration assessment. “Yes” is defined as positive symptom improvements in more than 3 weeks during the 6-week period; “no” is defined as positive symptom improvements in less than 3 weeks [18]. The indicator has been applied in several studies [19, 20] and was found to have significant correlations with intestinal function and QOL [21].

#### Secondary outcomes

##### IBS Symptom severity score

IBS symptom severity score (SSS) [22] is used to evaluate the degree of severity of IBS-D in the patients. A total score of 500 is calculated from abdominal pain degree, abdominal pain frequency, abdominal distension degree, defecation satisfaction, and influence on life. If the score is lower than 75, the patient is considered to be in remission. The mild, moderate, and severe boundary values are 75–175, 175–300, and above 300, respectively. The effectiveness, reliability, and sensitivity of IBS SSS to treatment are verified.

##### Bristol stool form scale

Grading standard in the Bristol stool form scale (BSS) [23] is employed to record stool characteristics of the patients with IBS-D in this study. The scale is a visual descriptive figure reflecting gastrointestinal transit time. The total score of the scale ranges from 1 to 7 according to the 1–7 types (from constipation to diarrhea) of stool characteristics.

##### Irritable bowel syndrome quality-of-life questionnaire

Irritable bowel syndrome quality-of-life questionnaire (IBS-QOL) is a specialized scale developed by Patrick et al. [24] for patients with IBS. It is composed of 34 items of self-assessment ranging from dysphoria, interference with activity, body image, health worry, food avoidance, social reaction, to sexual relationships, to assess QOL. The patients’ QOL is measured by a 5-point linear scale, and each question has a 5-point Likert scale (score 1–5). The total score of the scale ranges from 34 to 170. The scale achieves good measurement validity.

##### Hospital anxiety and depression scale

Hospital anxiety and depression scale (HADS) is used to assess the level of anxiety and depression in patients with IBS-D [25]. It comprises 14 questions, with seven questions each on anxiety and depression. Each question has a 4-point Likert scale (score 0–3). The total score of the scale ranges from 0 to 42. A score greater than 8 in each subscale indicates anxiety/depression.

IBS-SSS and BSS are completed before treatment (week 0), at the end of treatment (week 6), and during follow-ups (weeks 12 and 18). IBS-QOL and HADS are completed before treatment (week 0) and at the end of treatment (week 6). All the assessments are shown in Table 1.

**Table 1:** Trial flowchart

Items		Before enrolment (weeks) –4 to 0	Intervention period (weeks) 1–6	End of 6-week treatment (weeks)	Follow-up (weeks) 7–12	End of 12-week follow-up (weeks)	Follow-up (weeks) 13–18	End of 18-week follow-up (weeks)
Recruitment		×						
Enrolment		×						
Inclusion criteria		×						
Exclusion criteria		×						
Informed consent		×						
Basic characteristic variables		×						
Randomization and allocation concealment		×						
Primary outcomes	AR		×	×	×	×	×	×
Secondary outcomes	SSS	×		×		×		×
	BSS	×		×		×		×
	IBS-QOL	×		×				
	HADS	×		×				
Safety outcome				×		×		×
Statistical analysis				×		×		×

### Safety outcome

In this study, possible adverse events during moxibustion include skin burns, blisters, pruritus, dizziness, and respiratory symptoms. The researchers record and deal with all the adverse events (no matter whether it is relevant with moxibustion). When necessary, the researchers can decide whether the trial is terminated.

### Quality control

All the researchers are practicing doctors certified by the Ministry of Health of the People’s Republic of China. They must have more than 5 years of clinical experience and be qualified as attending physicians. All the researchers are trained to improve consistency among subjects. The outcomes are assessed for all the subjects. The outcome measurement data are preserved in the original case report form (CRF), and supervised and checked by a qualified clinical research coordinator. The original data are recorded into the electronic medical records using a double data entry method by trained researchers, and the electronic data are consistent with the original data. To improve compliance of the subjects, each subject who completes the trial is provided a cash reward.

### Data monitoring

This study has set up an independent clinical trial data monitoring committee (DMC), which comprises a medical expert and two clinical research experts. The DMC verifies whether the trial follows the study design and the standard guidelines, checks research progress and the accuracy and authenticity of the CRF, and checks whether the reasons for discontinuation of subjects and adverse events have been recorded in detail in the CRF. In the case of any serious adverse events, the DMC discusses with the institutional review board and the principal investigator, and the principal investigator makes a final decision on the continuation or termination of the trial. In addition, the institutional review board also makes recommendation to the principal investigator on stopping the trial if a serious adverse event occurs.

### Statistical analysis

The statisticians are responsible for coordinating research efforts, making a statistical analysis plan, and building a research database. Both intention-to-treat and per-protocol analyses are performed. The last-observation-carried-forward method is used for missing data in the intention-to-treat analysis. SPSS16.0 software is used to analyze the data. Measurement data are first tested using the normality test. Data conforming to the normal distributions are expressed as mean ± standard deviation, while data not conforming to the normal distributions are expressed as M (P25–P75). A paired *t* test or nonparametric test is used to perform intragroup comparison, and a two-sample *t* test or nonparametric test is used to perform comparison among groups. Enumeration data is expressed as a case (ratio) and analyzed using the *χ* ^2^ test. Statistic significance is defined as *p* < 0.05.

## Discussion

The present study assesses the efficacy and safety of mild-warm moxibustion treatment in patients with IBS-D through a multi-center, randomized, single-blind and placebo-controlled trial.

To confirm the efficacy of mild-warm moxibustion, placebo moxibustion is used as control which is included to eliminate potential placebo effect of moxibustion treatment. Methods for designing a placebo control group for mild-warm moxibustion were limited in the past. One study [26] used a 0.5 cm diameter moxa cone as a placebo in a blind trial. However, this method is impractical in our research design because the moxa stick used for warm moxibustion has to be longer than 1.5 cm which is also the most widely used specification in clinical practice.

At least two conditions should be satisfied in the design of placebo-controlled moxibustion: one is that the placebo model has no specific therapeutic function, the other is that subjects cannot perceive the difference between the placebo moxibustion and the real one. It has been proved that thermal stimulation is an important factor that influences the treatment efficacy, as the heat must reach a certain level to exert the effect [27, 28]. In this study, we use a moxa stick of 3 cm diameter to achieve better efficacy through stimulating the acupuncture points for more heat. Due to the unique nature of moxibustion treatment, if the heat stimulus was fully shielding, the participants will not be well blinded. Therefore, we try to reduce the heat stimulation of moxibustion to a minimum, while blind to the participants. The distance of the moxa stick to the acupuncture points in the placebo control group is set to 8–10 cm, and the temperature of the acupuncture point is maintained at 37 ± 1 °C. At this temperature, the subjects will feel “lukewarm”, because the temperature of human skin is between 30 and 36 °C with the average temperature at 33.5 ± 0.5 °C. In contrast, the distance of the moxa stick to the acupuncture points in the treatment group is set to 3–5 cm, so that the subjects feel warm without any burning pain. The temperature of the acupuncture point is maintained at 43 ± 1 °C, which is shown to contribute to the efficacy of moxibustion treatment [29]. It is important to mention that the subjects will be enrolled with no history of acupuncture and moxibustion treatment, and are not aware of the manipulation of moxibustion. Furthermore, they will be treated in individual isolated rooms, which prevent them from communicating with each other. Hence, the environment is conducive to perform the trial.

A limitation of the trial is that the study cannot double-blind the physicians and subjects. Thus, potential subjective factors in the clinician’s approach to the task and possible awareness of the subjects about the treatment they receive may lead to bias of the study.

The present protocol may demonstrate moxibustion as a safe and effective treatment for patients with IBS-D. If the goals of the study are achieved, we will provide new evidence that moxibustion may be an effective therapy for treating IBS-D. The low-cost, easy-applicable treatment will reduce the financial burden for the patients and healthcare system, relieve the medical burden of hospitals and save medical resources.

### Trial status

The trial is currently in the recruitment phase. Participant recruitment started in April 2015, and is expected to end in December 2017.

## Abbreviations

AR: Adequate relief
BSS: Bristol stool form scale
CRF: Case report form
DMC: Data monitoring committee
HADS: Hospital anxiety and depression scale
IBS: Irritable bowel syndrome
IBS-D: Diarrhea-predominant irritable bowel syndrome
IBS-QOL: Irritable bowel syndrome quality-of-life
IBS-SSS: Irritable bowel syndrome symptom severity score
TCM: Traditional Chinese medicine

## References

[1] Keszthelyi D, Troost FJ, Masclee AA (2012). Irritable bowel syndrome: methods, mechanisms, and pathophysiology. Methods to assess visceral hypersensitivity in irritable bowel syndrome. Am J Physiol Gastrointest Liver Physiol.

[2] Chey WD, Kurlander J, Eswaran S (2015). Irritable bowel syndrome: a clinical review. JAMA.

[3] Lovell RM, Ford AC (2012). Global prevalence of and risk factors for irritable bowel syndrome: a meta-analysis. Clin Gastroenterol Hepatol.

[4] Soubieres A, Pimentel M, Purdy C, Magar R (2015). Inclusion of a novel IBS blood panel for diagnosing diarrhea predominant irritable bowel syndrome (IBS-D): a UK perspective. Value Health.

[5] Camilleri M (2001). Management of the irritable bowel syndrome. Gastroenterology.

[6] Sandler RS, Everhart JE, Donowitz M, Adams E, Cronin K, Goodman C (2002). The burden of selected digestive diseases in the United States. Gastroenterology.

[7] Basilisco G (2007). Pathogenesis of irritable bowel syndrome: current understanding. Recenti Prog Med.

[8] Spiller R, Aziz Q, Creed F, Emmanuel A, Houghton L, Houghton L (2007). Guidelines on the irritable bowel syndrome: mechanisms and practical management. Gut.

[9] Li CY, Li SC (2015). Treatment of irritable bowel syndrome in China: A review. World J Gastroenterol.

[10] Grundmann O, Yoon SL (2014). Complementary and alternative medicines in irritable bowel syndrome: an integrative view. World J Gastroenterol.

[11] Magge SS, Wolf JL (2013). Complementary and alternative medicine and mind-body therapies for treatment of irritable bowel syndrome in women. Womens Health (Lond Engl).

[12] Park JW, Lee BH, Lee H (2013). Moxibustion in the management of irritable bowel syndrome: systematic review and meta-analysis. BMC Complement Altern Med.

[13] Ma YX, Liu X, Liu CZ, Wang LP, Gou G, Wang ZL (2013). Randomized clinical trial: the clinical effects of herb-partitioned moxibustion in patients with diarrhoea-predominant irritable bowel syndrome. Evid Based Complement Alternat Med.

[14] Longstreth GF, Thampson WG, Chey WD, Houghton LA, Mearin F, Spiller RC (2006). Functional bowel disorders. Gastroenterology.

[15] Qi L, Li N, Liu HR, Ma XP, Wu LY, Wang XM (2010). Clinical and experimental studies on moxibustion for treatment of irritable bowel syndrome. Chin J Tradit Chin Med Pharm.

[16] Kaptchuk TJ, Delley JM, Conboy LA, Davis RB, Kerr CE, Jacobson EE (2008). Components of placebo effect: randomized controlled trial in patients with irritable bowel syndrome. BMJ.

[17] General Administration of Quality Supervision, Inspection and Quarantine of the People’s Republic of China, Standardization Administration of the People’s Republic of China (2006). Nomenclature and location of acupuncture points (GB/T 12346-2006).

[18] Camilleri M, Mayer EA, Drossmana A, Heath A, Dukes GE, McSorley D (1999). Improvement in pain and bowel function in female irritable bowel patients with alosetron, a 5-HT3 receptor antagonist. Aliment Pharmacol Ther.

[19] Lembo A, Weber HC, Farraye FA (2003). Alosetron in irritable bowel syndrome: strategis for its use in a common gastrointestinal disorder. Drugs.

[20] Nyhlin H, Bang C, Elsborg L, Silvennoinen J, Holme I, Rüegg P (2004). A double-blind, Placebo-controlled, randomized study to evaluate the efficacy, safety and tolerability of tegaserod in patients with irritable bowel syndrome. Scand J Gastroenterol.

[21] Mangel AW, Hahn BA, Heath AT, Northcutt AR, Kong S, Dukes GE (1998). Adequate relief as an endpoint in clinical trials in irritable bowel syndrome. J Int Med Res.

[22] Francis CY, Morris J, Whorwell PJ (1997). The irritable bowel severity scoring system: a simple method of monitoring irritable bowel syndrome and its progress. Aliment Pharmacol Ther.

[23] Lewis SJ, Heaton KW (1997). Stool form scale as a useful guide to intestinal transit time. Scand J Gastroenterol.

[24] Patrick DL, Drossman DA, Frederick IO, Dicesare J, Puder KL (1998). Quality of life in persons with irritable bowel syndrome: development and validation of a new measure. Dig Dis Sci.

[25] Zigmond AS, Snaith RP (1983). The hospital anxiety and depression scale. Acta Psychiatr Scand.

[26] Zhao B, Wang X, Lin Z, Liu R, Lao L (2006). A novel sham moxibustion device: a randomized, placebo-controlled trial. Complement Ther Med.

[27] Pach D, Brinkhaus B, Willich SN (2009). Moxa sticks: thermal properties and possible implications for clinical trials. Complement Ther Med.

[28] Yi SH (2009). Thermal properties of direct and indirect moxibustion. J Acupunct Meridian Stud.

[29] Kim SY, Yi SH, Cho JH, Yin CS, Lee H, Park HJ (2011). Heat stimulation on the skin for medical treatment: can it be controlled?. J Altern Complement Med.

